# Metabolic relation of cyanobacteria to aromatic compounds

**DOI:** 10.1007/s00253-018-9568-2

**Published:** 2018-12-22

**Authors:** Beata Żyszka-Haberecht, Emilia Niemczyk, Jacek Lipok

**Affiliations:** Department of Analytical and Ecological Chemistry, Faculty of Chemistry, University of Opole, Oleska 48, 45-052 Opole, Poland

**Keywords:** Cyanobacteria, Aromatic compounds, Biotransformation, Metabolic relations, Metabolic response

## Abstract

Cyanobacteria, also known as blue-green (micro)algae, are able to sustain many types of chemical stress because of metabolic adaptations that allow them to survive and successfully compete in a variety of ecosystems, including polluted ones. As photoautotrophic bacteria, these microorganisms synthesize aromatic amino acids, which are precursors for a large variety of substances that contain aromatic ring(s) and that are naturally formed in the cells of these organisms. Hence, the transformation of aromatic secondary metabolites by cyanobacteria is the result of the possession of a suitable “enzymatic apparatus” to carry out the biosynthesis of these compounds according to cellular requirements. Another crucial aspect that should be evaluated using varied criteria is the response of cyanobacteria to the presence of extracellular aromatic compounds. Some aspects of the relationship between aromatic compounds and cyanobacteria such as the biosynthesis of aromatic compounds, the influence of aromatic compounds on these organisms and the fate of aromatic substances inside microalgal cells are presented in this paper. The search for this information has suggested that there is a lack of knowledge about the regulation of the biosynthesis of aromatic substances and about the transport of these compounds into cyanobacterial cells. These aspects are of pivotal importance with regard to the biotransformation of aromatic compounds and understanding them may be the goals of future research.

## Introduction

Cyanobacteria, which are microorganisms known as blue-green (micro)algae, are one of the phylogenetically oldest lineages and have continuously existed in the biosphere of the Earth for billions of years. These organisms are thought to be responsible for the gradual increase in the concentration of oxygen in the earth’s atmosphere and the so-called great oxygen event; they irreversibly changed the biosphere, allowing the expansion of oxygen-based life. Cyanobacteria are gram-negative bacteria, and they were the first organisms that were able to absorb solar light energy and transform it into chemical bond energy through the controlled flow of electrons in specifically adapted photosystems. According to the theory of endosymbiosis, this absolutely unique feature, photosynthesis, is a generous gift offered by the blue-green algae to the whole plant kingdom.

Cyanobacteria form a broad and diverse polyphyletic group of unicellular or simple multi-cellular microorganisms that are typically found in marine and freshwater environments. Nevertheless, these microalgae can also grow in many terrestrial environments, including caves, rocks and soil, and therefore are continually exposed to diverse natural and anthropogenic stressors. To overcome changes in the environment in which they live, cyanobacteria have developed a wide range of sensory mechanisms and adaptive systems. As pioneering organisms, blue-green algae have developed biochemical processes that allow them to acquire or synthesize all the chemicals essential for their structure and metabolism. This fascinating variety of natural products includes aromatic compounds.

The aim of this paper is to discuss the metabolism of aromatic compounds in cyanobacteria, including the biosynthesis of these chemicals, their influence on these organisms and the fate of the substances that reach microalgal cells. Such a holistic treatment of these specific topics has not been discussed previously.

## Biosynthesis of natural aromatic compounds by cyanobacteria

Aromatic compounds, which are widespread in the environment, come from natural, biological sources or result from anthropogenic activities. These unsaturated molecules consist of one or more rings (Favre and Powell 2013) that are in the simplest structural version, cyclic polyenes possessing conjugated π-electron systems of double bonds, meeting the Hückel rule. In accordance with the Molecular Orbital Theory, the “p” electrons on each sp^2^-hybridized carbon atom or heteroatom (nitrogen, oxygen or sulphur) are delocalized and contribute to the π system (Bruice 2004). The planar aromatic ring(s) provides structural and chemical stability due to the symmetrical π-electron systems and therefore contributes to the recalcitrance of these compounds (Vogt et al. 2011) and the specific reactivity of those systems. The matter of reaction specificity is especially important regarding heterocyclic compounds, which have one or more carbon atoms of the ring displaced by atom(s) of nitrogen, oxygen or sulphur. In the case of heterocyclic aromatics, the electron density is shifted towards the centre of the π-electron system proportional to the electronegativity of heteroatom(s), which creates, e.g. the possibility for electrophilic substitution at adjacent carbon atoms. For example, in the pyridine ring, the non-bonded electron pair located at the nitrogen atom causes a shift in π-electrons, which determines the reactivity of this substance. Although another heterocyclic nitrogenous aromatic base, adenine, which as a component of nucleic acids, is a component of all known living beings, microorganisms and plants are thought to be the primary producers of a variety of natural aromatic compounds.

**Fig. 1:**
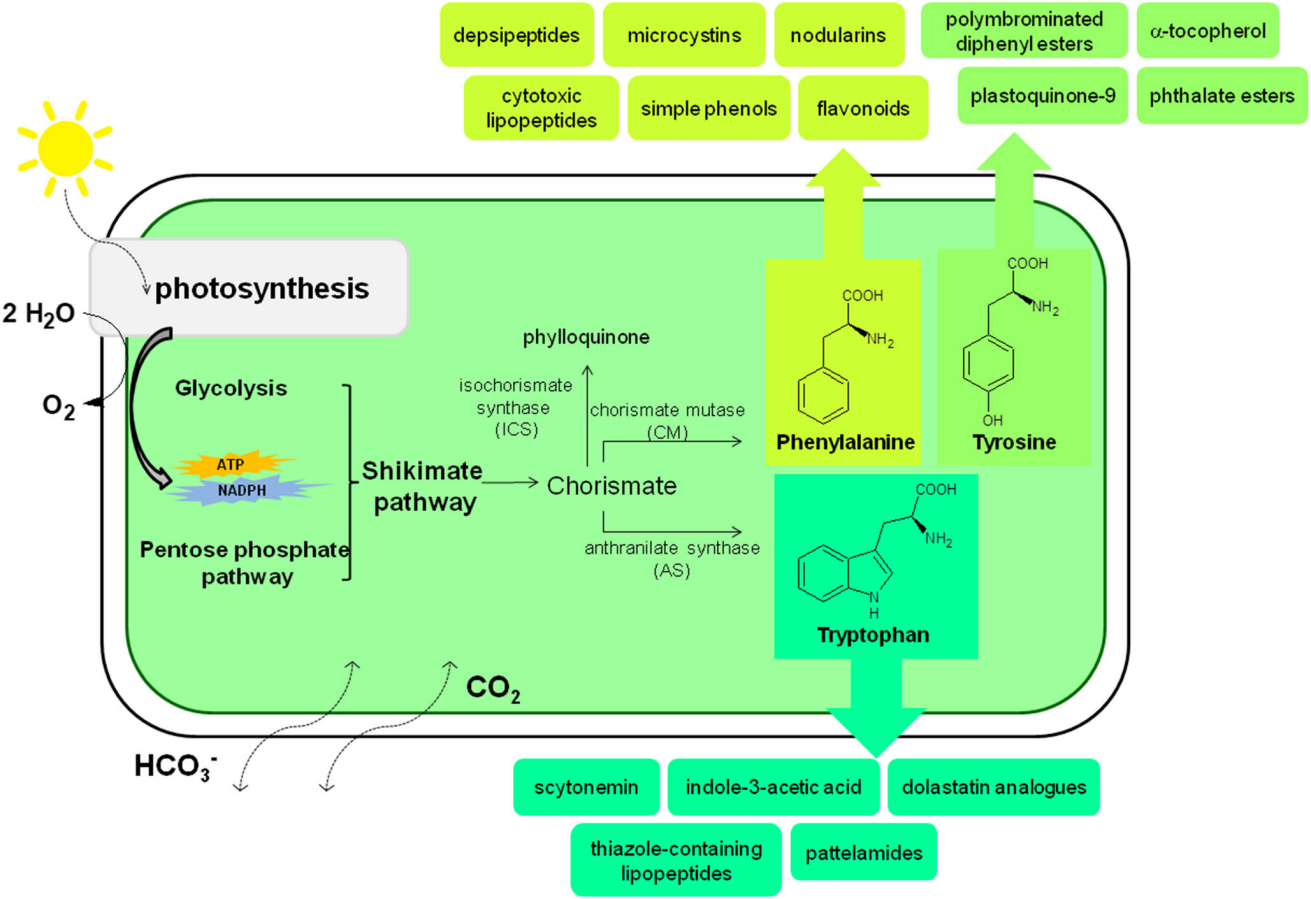
Biosynthesis of aromatic compounds by cyanobacteria based on cited literature

Cyanobacteria exhibit endo-oriented control of the multibranched aromatic biosynthetic pathway and synthesize the aromatic amino acids phenylalanine, tyrosine and tryptophan (Hall et al. 1983), which are the precursors for a large variety of secondary metabolites with aromatic ring structures. Cyanobacterial cells produce moderate amounts of aromatic amino acids under natural conditions (Dempo et al. 2014). Although the biosynthetic pathways of most cyanobacterial secondary metabolites are not well known, the chemical structures of representatives of those chemical junctions suggest that aromatic amino acids are their precursors (Fig. 1). Cyanobacterial species possess thylakoids, an intracellular membrane system that makes them highly suitable for the expression of the P450 enzymes that participate in phenylpropanoid biosynthesis (Melis 1999; Xue and He 2015). Phenolic compounds, especially simple phenols, cinnamic and 4-coumaric acid, are crucial precursors in the synthesis of various bioactive phenols (usually polyphenols) in photosynthetic organisms. The biosynthesis of cinnamic acid from phenylalanine is controlled by l-phenylalanine ammonia-lyase, a common plant enzyme, which was also found in cyanobacteria and was described for the first time in 2007 (Moffitt et al. 2007; Onofrejová et al. 2010). Various groups of benzoic acid derivatives (4*-*hydroxybenzoic, protocatechuic, gallic, vanillic and syringic acid), hydroxybenzaldehydes (4-hydroxybenzaldehyde and 3,4-dihydroxybenzaldehyde), cinnamic acid derivatives (2-coumaric, 4-coumaric, caffeic, ferulic, sinapic and chlorogenic acid), diaromatic phenolic compounds, (rutin, quercetin, apigenin, genistein, naringenin, acacetin and biochanin A) and flavonoids (dihydroquercetin, kaempferol and hyperoside) have been found in the biomass of halophilic and freshwater cyanobacteria (Klejdus et al. 2009; Onofrejová et al. 2010; Goiris et al. 2014; Babić et al. 2016; Żyszka-Haberecht et al. 2018). These results demonstrate that lower concentrations of phenols are synthesized in vivo by freshwater cyanobacteria than by freshwater algae.

Cyanobacteria are also able to synthesize the two types of quinines, phylloquinone (PhyQ) and plastoquinone-9 (PQ-9), that are synthesized exclusively by oxygenic phototrophs (Threlfall and Whistance 1971; Collins and Jones 1981); this exclusiveness results from the physiological functions of quinones being closely associated with oxygenic photosynthesis. Independent of the consensus suggesting that PQ-9 is probably synthesized via the conversion of chorismate to 4-hydroxybenzoate, which is then prenylated and decarboxylated (Pfaff et al. 2013), more recent studies suggest that cyanobacteria use both 4-hydroxybenzoate and homogentisate as the precursors of the PQ-9 head group (Nowicka and Kruk 2016).

Certain species of freshwater cyanobacteria produce phthalate esters, di(n-butyl)phthalate (DBP) and mono(2-ethylhexyl)phthalate (MEHP), which might affect neighbouring aquatic organisms. The type and quantity of these compounds depend on the species, displaying inter-generic, interspecific and intraspecific variations. Phthalate esters are de novo synthesized by cyanobacterial cells and are released into the environment under stressful conditions (Babu and Wu 2010b). Other studies demonstrated the occurrence of hydroxylated and methoxylated polybrominated diphenyl esters and polybrominated dibenzo-4-dioxins in the cells of cyanobacteria living in the Baltic Sea. Although the bioproduction of brominated compounds is relatively rare in filamentous cyanobacteria per se, due to the large production of biomass by these species, there may be considerable amounts of brominated aromatic compounds present in the Baltic Sea. The formation of brominated compounds in cyanobacteria has been suggested to occur via enzymatic bromination of organic substrates (Malmvärn et al. 2008), and bromoperoxidases were recently detected in some cyanobacterial species (Johnson et al. 2011).

Free-living cyanobacteria of various morphotypes, especially symbiotic isolates, can accumulate and/or release the phytohormone indole-3-acetic acid. Cyanobacterial accumulation of this auxin-like substance is stimulated by exogenous tryptophan and may proceed via the indole-3-pyruvic acid pathway (Sergeeva et al. 2002). Additionally, bloom-forming species of cyanobacteria are capable of producing structurally diverse bioactive metabolites containing mono- and diaromatic rings that are highly toxic to aquatic organisms, animals and humans (Pearson et al. 2010). Two of the four known classes of these cyanotoxins, microcystins and nodularins, have a unique C_20_ β-amino acid side-group with an aromatic functional group in their structures. The 3-amino-9-methoxy-2,6,8-trymethyl-10-phenyldeca-4,6-dienoic acid and other amino acids with diverse structures differ in various aspects, such as methylation, epimerization, hydroxylation, peptide sequence and toxicity. These cyanotoxins are non-ribosomally synthesized by the thiotemplate mechanism of a large multifunctional enzyme complex containing both non-ribosomal peptide synthetase and polyketide synthase domains. The gene cluster encoding these biosynthetic enzymes, mcyS and ndaS, has been sequenced and partially characterized in several cyanobacterial species (Pearson et al. 2010). Many natural aromatic products isolated from blue-green algae, such as alkaloids, cytotoxic lipopeptides, cyclic peptides, depsipeptides and patellamides, also contain nitrogen in their indole, imidazole, oxazole, thiazole, pyridine or quinoline functional groups (Folmer et al. 2010; Mo et al. 2010; Raveh and Carmeli 2007; Jin 2011; Orjala and Gerwick 1997). The UV-absorbing indole-alkaloid sunscreen pigment scytonemin, containing four aromatic rings, appears only in sheaths enclosing the cells of cyanobacteria. Scytonemin protects cyanobacterial cells by efficiently absorbing UV irradiation and dissipating this energy as harmless heat to the surroundings (Conde et al. 2004). Similarly to the aforementioned cytotoxic peptides, scytonemin shows anticancer activity (Folmer et al. 2010). Scytonemin was proposed to be biosynthesized from secondary metabolites by the condensation of tryptophan and phenylpropanoid-derived subunits (Proteau et al. 1993), and this process can be induced by a high photon fluence rate (Garcia-Pichel and Castenholz 1991).

Despite this progress, there are still unknown cyanobacterial pathways involved in the conversion of aromatic compounds. These pathways enable the recycling of aromatic compounds produced by the cells and the utilization of exogenous compounds (Nowicka and Kruk 2016). The biological functions of phenolic compounds in cyanobacterial cells also remain to be clarified, especially given that the low concentrations of these substances found in the cells of these microorganisms limit their role as antioxidants. Nevertheless, the prevalence of antioxidant polyphenols in cyanobacterial species is presumably an adaptation strategy against abiotic stresses (Singh et al. 2017).

## The response of cyanobacteria to the presence of aromatic compounds

In addition to biosynthesis, another important aspect of the relationship between cyanobacteria and aromatic compounds is the response of these organisms to the presence of these chemicals. The effect of aromatic compounds on microorganisms can be evaluated based on various criteria. For instance, to characterize the impact of any factor at the physiological level, the biomass dry weight or the chlorophyll *a*, phycobiliprotein, nucleic acid, protein or ATP concentration are often used as indicators. The activity of antioxidant enzymes or other enzymes involved in important metabolic reactions is also often measured. The responses to stress caused by aromatic compounds in cyanobacteria and other microalgae have been extensively reviewed over the years because these molecules, such as benzene derivatives and polycyclic aromatic hydrocarbons are often used as pesticides (herbicides and insecticides) and are significant environmental pollutants (Ramakrishnan et al. 2010; Singh and Khattar 2016; Kaushik et al. 2018a, b; Singh et al. 2018; Kumar et al. 2018).

Phenol and its derivatives are one of the largest groups of environmental pollutants due to their presence in many industrial effluents (such as those from oil and petroleum refineries: coking plants; coal processing; resin, paint, dye, and petrochemical production; and textile and paper mills) and their broad application as antibacterial and antifungal agents (Zhang et al. 2013; Krastanov et al. 2013). Shashirekha et al. (1997) reported that the application of 25 and 50 mg/L phenol decreased the chlorophyll content of *Phormidium valderianum* compared to that of control treatments. However, cyanobacterium show increased protein content in response to phenolic exposure. The influence of polyphenols (gallic acid (GA), pyrogallic acid (PA), catechol (CA), tannic acid (TA), pyrogallol (PYR), and hydroquinone (HQ)) on the metabolic response of *Microcystis aeruginosa* has been investigated using various methods. Ni et al. (2013) focused on changes in growth rate, enzymatic antioxidants (superoxide dismutase (SOD) and catalase (CAT)) and concentrations of soluble protein and sugar under exposure to GA, PA and CA. All three polyphenols tested inhibited growth. Interestingly, the presence of GA and CA (25 mg/L) stimulated the production of protein and sugar as well as the activation of SOD and CAT. Laue et al. (2014) reported that the application of GA and TA (more than 1 mg/L) inhibited growth (as measured by biomass and on chlorophyll *a* content) and the efficiency of photosystem II in *M*. *aeruginosa.* Dziga et al. (2007) showed that PYR and HQ influence the activity of alkaline phosphatase and photosynthesis. These polyphenols probably induce growth restriction by negatively impacting both the activity of enzymes and photosynthetic processes.

The effects of aromatic industrial pollutants, such as benzene, toluene, xylene and 4-nitrophenol, on various cyanobacterial strains have also been evaluated. Sundaram et al. (2011) showed that stressors used at concentrations up to 300 μM caused changes in the structures of *Nostoc muscorum* and *Synechococcus* PCC7942 cells. Among the organic stressors investigated, xylene inhibited the growth (in terms of specific growth rate) of both microorganisms the most. Toluene caused a similar effect as xylene but only in the case of *Synechococcus* cells. Both benzene and *para*-nitrophenol had a mild influence on the growth rate of the cyanobacteria. The impact of benzene and its derivatives on *Arthrospira platensis* and *Anabaena cylindrica* has also been studied, and all examined organic solvents were highly toxic to the halophilic cyanobacterium. Different results were obtained with respect to the freshwater *A. cylindrical*; in this case, 300 μM benzene, toluene and xylene significantly reduced the growth rate, while *para*-nitrophenol, even at a higher dose (400 μM), affected the growth to a lesser extent (Sundaram and Soumya 2011).

Another group of aromatic environmental pollutants are the phthalate esters (PEs), which are well known because of their broad use as plasticizers. Acey et al. (1987) reported an inhibitory effect of di-n-butyl phthalate (DBP) on the growth rate of *Synechococcus lividus* (estimated using haemocytometry). Moreover, DBP promotes cell aggregation. Babu and Wu (2010a) examined the influence of DBP, diethyl phthalate (DEP) and dimethyl phthalate (DMP) on the growth (based on the absorbance at 663 nm) of three cyanobacteria: *Anabaena flos*-*aquae*, *Microcystis aeruginosa* 2396 and *Microcystis aeruginosa* 4141. All tested PEs at all tested concentrations (20, 30, 50 mg/L for DBP and 100, 150, 200 mg/L for DMP and DEP) increased the growth of the tested microorganisms in a concentration-dependent manner. The impact of DMP on the growth rate (based on the absorbance at 730 nm) was also studied in three other cyanobacteria: *Synechocystis* sp., *Synechococcus* sp. and *Cyanothece* sp. The lowest applied concentrations of DMP (20 mg/L) induced growth-promoting activity, whereas higher doses (50–500 mg/L) inhibit cyanobacterial growth (Zhang et al. 2016).

Di-, tri- and polycyclic aromatic hydrocarbons are commonly found toxicants; nevertheless, naphthalene and anthracene at a concentration of 0.05% (*w*:*v*) enhanced the growth (based on protein and chlorophyll *a* concentrations) of the saline cyanobacterium *Phormidium tenu.* In contrast, the growth of other tested *Phormidium* strains was suppressed*.* Higher concentrations of both aromatic hydrocarbons were lethal for all tested species (Kumar et al. 2009). Huang et al. (2016) studied the interactions between diaromatic triclosan (TCS) and the cyanobacteria *Microcystis aeruginosa*. The results showed that even 1 μg/L of this chemical inhibited growth and that a higher dose (10 μg/L) induced deactivation of photosynthesis. Exposure to TCS caused changes in SOD activity, r educed glutathione content and cellular ultrastructure. Patel et al. (2015) measured the effect of anthracene on chlorophyll *a*, carotenoids and phycobiliproteins in three different cyanobacterial strains, *Synechocystis* sp., *Anabaena fertilissima* and *Nostoc muscorum*. This study monitored the changes in the content of pigments, carbohydrates, proteins, amino acids and phenols during exposure to anthracene. A dose-dependent reduction in the concentrations of those metabolites, except for phenols, was observed for all tested cyanobacteria. The influence of pyrene, a polyaromatic hydrocarbon pollutant of growing concern, was investigated in a similar way in *Synechocystis* sp., *Anabaena fertilissima* and *Nostoc muscorum.* In this case, a comparable response to that of anthracene was obtained (Patel et al. 2013; Kumar et al. 2014). Very recently, Zhang et al. (2018) studied the effects of pyrene and naphthalene on the growth rate, phycobiliprotein concentration, microcystin content and antioxidant enzyme activity of *Microcystis aeruginosa*. The results of those experiments revealed that the growth of the cyanobacterium was modified and the phycobiliprotein content was decreased, whereas the production of a microcystin-specific toxin was promoted by the presence of the tested chemicals. *M. aeruginosa* has also been the subject of studies on the effect of *N*-phenyl-1-naphthylamine (P1NA) and *N*-phenyl-2-naphthylamine (P2NA) on the growth rate (based on cell density), photosynthetic rate, esterase activity and cell membrane integrity (Cheng et al. 2017). P2NA is less hazardous to cells than P1NA. The growth inhibition and membrane damage caused by exposure to P1NA are proportional to the concentration. The esterase and photosynthetic activity of *M. aeruginosa* was reduced to 22.2 and 3.3%, respectively, when its cells were exposed to 20 μM P1NA. In the presence of 20 μM P2NA, these activities were only slightly reduced (approximately 3–8%).

The continuous exposure of cyanobacteria to the aromatic compounds used as pesticides and the interactions between these biota and chemicals are known to be the best-explored area regarding these metabolic interactions. The influence of carbamate insecticides (monoaromatic carbaryl and diaromatic carbofuran) was tested in different nitrogen-fixing cyanobacterial species. Vaishaampayan (1985) reported that 120 μg/mL carbaryl completely inhibits the growth of *Nostoc muscorum*, whereas approximately 100 ppm of this substance was lethal for *Cylindrospermum* sp. in nitrogen-fixing medium (C-N) and medium with nitrate supplementation (C+N). Interestingly, 25 ppm carbofuran stimulates the growth of *Cylindrospermum* sp. in both conditions. Lethality was observed at 2000 and 3000 ppm under C-N and C+N conditions, respectively (Panigrahy and Padhy 2000). The toxic effects of carbaryl and carbofuran on another N_2_-fixing cyanobacterium, *Anabaena* PCC 7120, have also been investigated. The growth of this microorganism is completely suppressed by carbaryl at 100 and 120 ppm under nitrogen-fixing conditions and nitrate supplementation, respectively. Carbofuran is less toxic to cyanobacterial cells: only concentrations of 1500 ppm or higher are lethal (Padhy 2001; Padhy and Mohapatra 2001). Dash et al. (2017) reported on the comparative influences of insecticides (carbofuran, quinalphos and methyl parathion) and herbicides (butachlor, benthiocarb and 2,4-D) on the growth, acetylene reduction activity (ARA) and N-yield of native cyanobacteria in experimental rice fields (belonging to the genera *Anabaena*, *Aphanothece*, *Aulosira*, *Gloeotrichia* and *Nostoc*). These parameters were higher than the control levels in the experiments with carbofuran and methyl parathion; however, quinalphos caused a reduction in these parameters compared with those of the control. Among the herbicides, butachlor and benthiocarb were harmful to the examined species, whereas 2,4-D resulted in an increase in growth, ARA and N-yield. Kaushik et al. (Kaushik et al. 2018a, b) observed that 0.5 ppm benthiocarb did not affect the growth (in terms of protein content) and reduced the acetylene reduction activity of *Nostoc linckia.* However, the growth and ARA of *Aphanothece pallida* in the presence of this chemical was stimulated. Interestingly, another tested pesticide, methyl parathion, at 0.25 ppm resulted in faster growth and increased ARA in both species. Tiwari et al. (2018) observed changes in growth, phycobiliprotein and carbohydrate contents, and protein- and gene-expression patterns in *Fischerella* sp. under methyl parathion stress. The growth of the cyanobacterium, photosynthetic efficiency, and the content of chlorophyll and phycobiliproteins were all reduced. However, methyl parathion caused the induction of antioxidative enzymes, chaperones, signalling proteins and peptidases, ensuring survival.

**Fig. 2:**
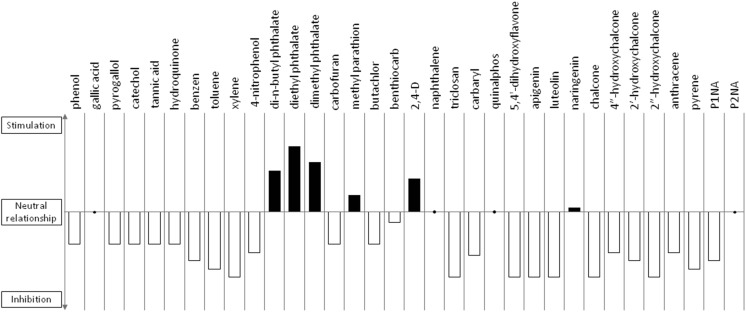
An overview of the impact of aromatic compounds discussed in the text on the growth of cyanobacteria. The stimulatory or inhibitory effect was estimated on the basis of the average response of various strains of microalgae to the presence of xenobiotics. The values above the *x*-axis indicate growth stimulation, and the values below indicate growth suppression in the presence of chemicals. The neutral relationship is shown with dots

Little information is available describing the interaction between non-pollutant aromatic compounds and cyanobacteria. Noted examples are related to the extracts of various aquatic and terrestrial plants that contain biologically derived substances (BDSs) that are able to inhibit the growth of prokaryotic microalgae. The anti-algal BDSs that have been identified are mainly phenols, alkaloids, terpenes, organic acids, flavonoids and others (Shao et al. 2013). Investigations into cyanobacteria-flavonoid interactions have been accomplished using 5,4'-dihydroxyflavone (DHF), apigenin and luteolin in *Microcystis aeruginosa.* A decrease in cyanobacterial growth occurred even at the lowest concentrations of flavonoids examined (0.1 mg/L). DHF and apigenin induce slight negative effects on the photosynthetic activity, whereas luteolin significantly disturbs the activity of the photosystems. Flavonoids influence the growth of cyanobacteria by changing the integrity of the cellular membrane (Haung et al. 2015). The impact of naringenin on three halophilic *Arthrospira* strains and five freshwater strains (*Anabaena* sp., *Anabaena laxa*, *Nodularia moravica, Chroococcus minutus and Nostoc muscorum*) has also been studied. The application of naringenin at micromolar levels (5–10 mg/L) caused stimulation of growth (based on chlorophyll content), especially with *N. muscorum*. In the case of halophilic species, the stimulation of growth was observed when the concentration of the chemical was below 40 mg/L. Higher levels of naringenin (above 40 mg/L) were found to be harmful to the microorganisms examined. Interestingly, the presence of naringenin not only influences the growth of cyanobacteria but also changes the permeability of the cell walls and cellular membranes in a dose-dependent manner, but these effects depend on the cyanobacterial strain (Żyszka et al. 2017b). The influence of chalcone at 20 mg/L on the growth of *Aphanizomenon klebahnii* has also been described (Żyszka et al. 2017a). The results showed that in the presence of chalcone, the growth of the tested cyanobacteria is markedly slower than that of the control. This study also examined the impact of hydroxylated chalcones (4″-hydroxychalcone, 2′-hydroxychalcone and 2″-hydroxychalcone at 20 mg/L) on one halophilic strain (*Arthrospira platensis*) and seven freshwater strains (*Anabaena* sp*., A. laxa*, *A. klebahnii*, *N. moravica*, *Ch. minutus*, *Merismopedia glauca*, and *Synechocystis aquatilis*). All hydroxylated chalcones tested inhibited the growth of the tested strains of cyanobacteria. Among them, 2″-hydroxychalcone was found to elicit the strongest negative effect, whereas 4″-hydroxychalcone affected cyanobacterial growth the least. *A. klebahnii*, whose growth was suppressed completely even by 4″-hydroxychalcone, was found to be the most sensitive strain (Żyszka-Haberecht et al. 2018). The influence of aromatic compounds on cyanobacteria may be summarized as the following: in addition to esterified phthalates, which are structurally recognized as being naturally produced by certain species of freshwater green-blue algae and thus promoting their growth, most of the examined aromatic substances inhibit the growth and development of microalgae in a dose-dependent manner (Fig. 2).

## The fate of aromatic compounds in cyanobacterial cells

### Biotransformation of monoaromatic hydrocarbons by cyanobacteria

Some species of cyanobacteria are capable of degrading industrial pollutants, such as benzene, toluene and xylene (Sundaram and Soumya 2011). One way of investigating the biodegradation of organic pollutants by cyanobacteria is to encourage the cells to tolerate and to grow in the presence of the pollutant. An example is the marine cyanobacterium *Phormidium valderianum*, which is able to grow in the presence of phenol and simultaneously remove it from the culture medium through the intracellular activity of polyphenol oxidase, as well as of laccase enzymes (Shashirekha et al. 1997). Samanthakamani and Thangaraju (2015) indicated that phenol is susceptible to decomposition by other blue-green algae species of the genera *Anabaena*, *Nostoc*, *Oscillatoria* and *Spirulina*, which can use phenol to meet their needs for a carbon source and energy (Samanthakamani and Thangaraju 2015). Another interesting observation was in a study by Wurster et al. (2003), in which another taxon, *Synechococcus* PCC 7002, was shown to be capable of degrading phenol under non-photosynthetic conditions in darkness, but the cells were not able to use phenol for growth. This activity leads to the formation of cis-, cis-muconic acid, which is formed by the ring cleavage of the compound, providing the first record of the ortho-fission of a phenolic substance by cyanobacteria. Further investigations into phenol breakdown mechanisms have shown that the transformation is an extracellular process that is inhibited by heat, proteases and metal ions and is probably catalyzed by a protein (Wurster et al. 2003). Another study demonstrated the potential for cyanobacterial exudates to affect the metabolism of heterotrophic bacterial degraders, particularly in pulp and paper waste treatment systems. Cyanobacterial exudates provide both an endogenous source of growth substrates to bacteria and influence the rate of degradation of key aromatic contaminants. As an example, biodegradation results showed that phenol removal and *Pseudomonas* sp. growth are significantly lower in the presence of exudates from three of the four exudate sources than in the control but were slightly stimulated by exudates from *Pseudanabaena* PP16 (Kirkwood et al. 2006).

Two cyanobacterial strains, *Agmenellum quadruplicatum and Oscillatoria* sp. grown photoautotrophically in the presence of aniline metabolized the aromatic amine into formanilide, acetanilide and 4-aminophenol, wherein the *N*-formylated and *N*-acetylated derivatives of aniline were the major metabolites. Because cyanobacteria metabolize aniline predominantly to metabolites that are less toxic than the parent compound, it seems that the enzymatic capacity to detoxify aromatic amines may have evolved in oxygenic photosynthetic prokaryotes (Cerniglia et al. 1981).

Interactions between nitrobenzene and *Microcystis aeruginosa* revealed that only a small amount of nitrobenzene can be adsorbed by this strain, and the adsorption process allows the mentioned molecule to connect and enter into the cells of cyanobacteria. The results of cell activity and illumination experiments unequivocally confirmed that *M. aeruginosa* can reduce nitrobenzene to aniline by the activity of nitrobenzene reductase. Nevertheless, *M. aeruginosa* does not show the ability to degrade aniline and prevent its volatilization through the accumulation of this substance in the reaction medium (Liu et al. 2014).

2,4-dinitrophenol, which is widely used as a precursor in the production of pesticides, herbicides, and synthetic dyes, is often found as the xenobiotic in industrial wastewater (Hirooka et al. 2003; Hirooka et al. 2006). In an appropriate screening study, *Anabaena cylindrica* showed the greatest ability to remove low concentrations of 2,4-dinitrophenol and to reduce it to 2-amino-4-nitrophenol under the presence of light (Hirooka et al. 2006). Importantly, however, a mixed culture of two cyanobacterial species, *A. variabilis* and *A. cylindrica*, can remove not only the initial substrate but also the product of its degradation: 2-amino-4-nitrophenol. This ability is expected to be useful in the treatment of industrial wastewater (Hirooka et al. 2006).

The reductive transformation of 2,4,6-trinitrotoluene was observed in a continuous-flow system of *Anabaena* sp. (Pavlostathis and Jackson 2002). Effective 2,4,6-trinitrotoluene removal without toxic culture effects is feasible using a continuous-flow system. The only compounds detected in extracts of batch *Anabaena* sp. cultures that had transformed 2,4,6-trinitrotoluene were three azoxy-tetranitrotoluene isomers (2-2′ Azy-TeNT, 2-4′ Azy-TeNT, and 4-4′ Azy-TeNT), which, taken together, accounted for only 4.4% of the initial moles of 2,4,6-trinitrotoluene added to these cultures (Pavlostathis and Jackson 1999).

Due to the activity of 3-ketoacyl-(acyl-carrier-protein) reductase (Hölsch et al. 2008), the representatives of the few genera of cyanobacteria, *Nodularia*, *Arthrospira*, *Leptolyngbya*, and *Synechococcus*, efficiently transform prochiral acetophenone into two desired products, optically pure (R)- and (S)-1-phenylethanol, with high yields and good enantioselectivity (Głąb et al. 2016). Because both mentioned chiral aromatic alcohols are essential building blocks in the pharmaceutical and cosmetic industries (Cao et al. 2012), the biocatalytic conversion of acetophenone and its derivatives was treated as the model process of bioreduction. It has also been shown that this process depends more on the presence of glucose, which is used as a co-substrate for the regeneration of the cofactor NADPH in the medium, than on the light regime (Głąb et al. 2016). Interestingly, all tested cyanobacterial strains were more likely to form (S)-1-phenylethanol than (R)-1-phenylethanol. This phenomenon was also confirmed by the results of studies on biotransformation of fluoro-, chloro-, methyl- and *O*-methyl- derivatives of acetophenone, by *Synechococcus* sp. *Nostoc* sp., and *Synechocystis* sp. strains (Nakamura et al. 2000; Nakamura and Yamanaka 2002; Itoh et al. 2014).

Phthalate esters are environmental pollutants that are commonly used as plasticizers. Babu and Wu (2010a) shown that the degradation of phthalate esters occurs with di-n-butyl-, diethyl-, and dimethyl- substituents through transesterification on the side chains of phthalate esters in cyanobacterial species such as *Anabaena flos-aquae* and *M. aeruginosa*. Additionally, experiments showed that there are two degradation pathways of these biotransformation reactions: C16→C14→C12→C10→C8 and C16→C15→C13→C11→C9, which follow first-order kinetics (Babu and Wu 2010a).

The results of other experiments on the biodegradation characteristics of dimethyl phthalate (DMP) by freshwater unicellular organisms revealed that all the organisms analysed were capable of metabolizing DMP. Among them, *Cyanothece* sp. PCC7822 achieved the highest degradation efficiency. Low concentrations of DMP supported the growth of cyanobacteria; however, with increased DMP concentration, the growth of cyanobacteria was remarkably inhibited. Phthalic acid (PA) was determined to be an intermediate degradation product of DMP that accumulates in the culture solution. Additionally, esterase is induced by DMP, and the activity continually increases during the degradation process, suggesting that esterase can participate in the degradation of DMP (Zhang et al. 2016).

The incubation of whole cells of *Nostoc muscorum* with codeine under a continuous light photo regime yielded four transformation products: 6-acetylco-deine, oxycodone, norcodeine and morphine. The observed modifications included *O*-demethylation, *N*-demethylation, C6-acetylation, C14-hydroxilation, Δ^7^-reduction and C6-oxidation. The capacity of *N. muscorum* to convert codeine to oxycodone represents an uncommon pattern of codeine metabolism in microorganisms that may be of industrial importance (Niknam et al. 2010).

Cyanobacterial species are also able to transform an organophosphorus pesticide, fenamiphos, to its primary oxidation product, fenamiphos sulfoxide (FSO) and to hydrolyze FSO to fenamiphos sulfoxide phenol (FSOP). The ability of these cyanobacteria to detoxify fenamiphos can be advantageously used in the bioremediation of this pesticide and its toxic metabolites (Cáceres et al. 2008).

### Biotransformation of diaromatic hydrocarbons by cyanobacteria

**Fig. 3:**
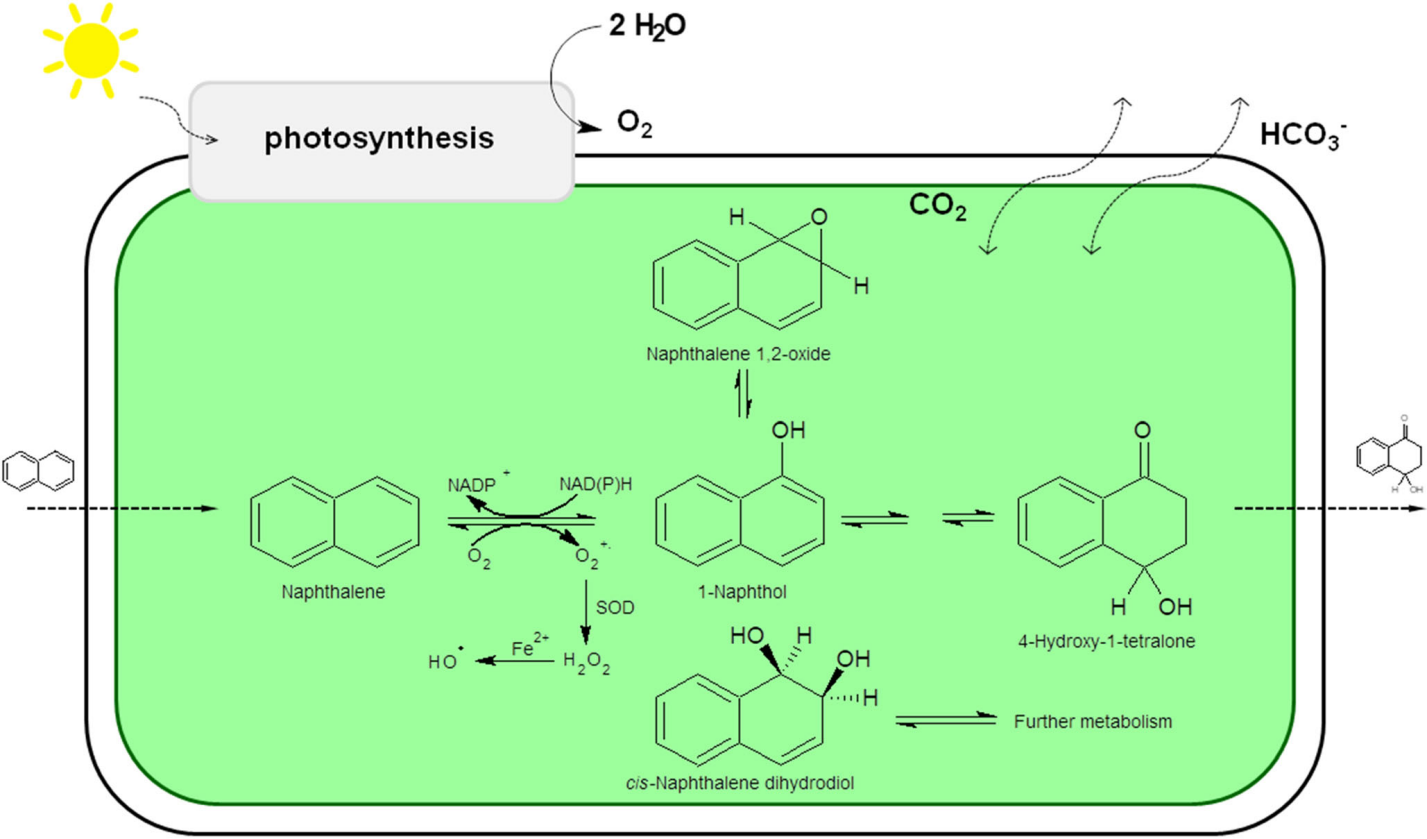
Proposed pathways of the transformation of naphthalene by *Oscillatoria* sp.

In the presence of naphthalene and under photoautotrophic growth, the cyanobacterial strain *Oscillatoria* sp. oxidizes this aromatic hydrocarbon to cis-1,2-dihydroxy-1,2-dihydronaphthalene, 4-hydroxy-1-tetralone and 1-naphthol. The major metabolite is 1-naphthol (Cerniglia et al. 1980a). Other blue-green algae of the genera *Anabaena*, *Agmenellum*, *Aphanocapsa*, *Coccochloris*, *Microcoleus* and *Nostoc* also oxidize naphthalene to 1-naphthol under photoautotrophic conditions (Fig. 3) (Cerniglia et al. 1980c).

The degradation of naphthalene by a hypersaline *Phormidium tenue* strain resulted in the formation of [1,2]naphthoquinone and naphthalene-1,2-diol presumably through an inducible enzyme system. These results suggest a possible role for a dioxygenase enzyme system in cyanobacterial degradation (Kumar et al. 2009). El-Sheekh et al. 2012 performed work on the oxidation of phenolic compounds, such as α-naphthol and β-naphthol. The compound α-naphthol was degraded to approximately 40% of the initial concentration by three cyanobacterial strains, *Lyngbya lagerlerimi*, *Nostoc linckia* and *Oscillatoria rubescens* after 5 days of incubation. The oxidation of β-naphthol by these cyanobacterial species is accompanied by a shift in wavelength and change in colour (El-Skeekh et al. 2012). Another *Oscillatoria* sp. can be grown photoautotrophically in the presence of biphenyl, and it oxidizes this compound predominantly at the 4-position to form 4-hydroxybiphenyl (Cerniglia et al. 1980b).

The results of the interactions between the antimicrobial agent triclosan (TCS) and the bloom-forming cyanobacteria *Microcystis aeruginosa* reveal that TCS can be transformed by this cyanobacterium, with methylation as the major biotransformation pathway. Furthermore, the presence of *M. aeruginosa* in solution promotes the photodegradation of TCS. Overall, *M. aeruginosa* plays an important role in the dissipation of TCS in aquatic environments through the sorption and biotransformation of cyanobacterial cells (Huang et al. 2016).

The light-catalyzed bioreduction of chalcone into the corresponding dihydrochalcone was observed using intact cells of selected cyanobacterial strains. *Anabaena laxa*, *Aphanizomenon klebahnii*, *Nodularia moravica* and *Synechocystis aquatilis* can efficiently catalyze this reaction (with a yield of over 99%). The transformation rate of the reported bioconversion of chalcone by *Merismopedia glauca* was slightly lower (92%). *Anabaena* sp. and *Chroococcus minutus*, apart from the dominant product (dihydrochalcone), formed several other metabolites (1,3-diphenyl-2-propen-1-ol, 1,3-diphenylpropan-1-ol, cinnamic acid, hydrocinnamic acid). Moreover, the scaled-up culture of *A. klebahnii* in a photobioreactor, under controlled light conditions, pH and temperature, resulted in improved reaction time (shortened by 2-fold), while the biotransformation rate remained unchanged (Żyszka et al. 2017a). Recently, Żyszka-Haberecht et al. (2018) also reported the bioconversion of hydroxychalcones by the same species of cyanobacteria as in previous studies. The biotransformation reactions of 2″-hydroxychalcone that were catalyzed by all tested cyanobacteria were extremely chemoselective, forming only 2″-hydroxydihydrochalcone, and were highly efficient. The yield of the conversions conducted by *A. laxa*, *Anabaena* sp., *A. klebahnii*, *N. moravica*, *Ch. minutus* and *S. aquatilis* was higher than 94%. Similarly, the most abundant product of the bioconversion of 4″-hydroxychalcone was 4″-hydroxydihydrochalcone, and in the case of the strains *A*. *laxa*, *Ch. minutus*, *M. glauca*, *S. aquatilis* and *S. platensis*, 4″-hydroxydihydrochalcone was the only metabolite formed. The fact that *Anabaena* sp., *A. klebahnii* and *N. moravica* carried out a biotransformation that yielded two other products, 4″-hydroxy-1,3-diphenylpropan-1-ol and 4″,x″-dihydroxydihydrochalcone, in addition to the main product, confirmed that the position of the hydroxyl substituent determines the type of bioconversion (hydrogenation and/ or hydroxylation) and its efficacy (Żyszka-Haberecht et al. 2018).

### Biotransformation of tri- and polycyclic aromatic hydrocarbons by cyanobacteria

The degradation of anthracene by the hypersaline *Phormidium tenue* strain showed the presence of anthracene-1,2-dione, 8-hydroxy-anthracene-1,2-dione and 10-hydroxy-anthracene-1,2-dione. Anthracene-1,2-dione is thought to be the first degradation product, which is then converted to 9-hydroxyanthracene-1,2-dione. Oxidation of aromatic ring carbon is an energy-providing process that often leads to complete degradation of the substrate (Kumar et al. 2009). *Anabaena fertilissima* can degrade anthracene to 2,4-dimethyl-1-heptene, 9-octadecane, tetradecanoic acid, and benzene ring compounds (Patel et al. 2015). Other experiments showed that individual cyanobacteria and cyanobacterial consortia effectively degraded anthracene, benzo(a)anthracene and dibenzo(a,h)anthracene without nutrient amendment or biological stimulation of the studied organisms implicated in the process (Ichor et al. 2017).

Phenanthrene, a tricyclic aromatic hydrocarbon, is widely distributed in the environment as a result of pyrolytic processes. Under photoautotrophic growth conditions, the marine cyanobacterium *Agmenellum quadruplicatum* PR-6 metabolized phenanthrene to form trans-9,10-dihydroxy-9,10-dihydrophenanthrene (phenanthrene trans-9,10-dihydrodiol) and 1-methoxyphenanthrene as the major ethyl acetate-extractable metabolites. Small amounts of phenanthrols were also formed. Oxygenation of the aromatic ring is catalyzed by a monooxygenase rather than a dioxygenase. Phenanthrene is metabolized by *Agmenellum quadruplicatum* as a detoxification reaction (Narro et al. 1992). Phenanthrene can be completely mineralized by cyanobacteria and a consortium of aerobic heterotrophic bacteria and cyanobacteria (Ichor et al. 2016).

*Anabaena fertilissima* can degrade pyrene by 33%. The common degraded product for pyrene is 2,3,4-trimethylhexane (Patel et al. 2015), whereas *Anabaena flos-aquae* metabolizes benzo(α)pyrene to dihydrodiols at a much slower rate than the green algal species (Warshawsky et al. 1995). The other triaromatic systems, *N*-phenyl-1-naphthylamine (P1NA) and *N*-phenyl-2-naphthylamine (P2NA), are both widely used antioxidants and plant secondary metabolites. Interestingly only P1NA is biotransformed to 1,4-naphthoquinone by *Microcystis aeruginosa*. It is worth noting that the formation of 1,4-naphthoquinone is a key factor in the generation of excessive reactive oxygen species (Cheng et al. 2017).

## Conclusions

The natural production of a wide range of secondary metabolites, including aromatic ones, is a common adaptation of cyanobacteria that allows them to successfully compete in a variety of ecosystems. Because of their phototrophic lifestyle and constant exposure to high oxygen and radical stresses, these organisms possess a high capacity for producing plentiful and efficient chemicals to protect against oxidative and radical stressors. This ability is especially important for cyanobacteria because the energy supply, the key factor that enables chemical transformations in cellular systems, naturally induces the formation of radicals. In the case of oxygenic photoautotrophic organisms, the solar light energy captured by photosynthetic systems is converted to electrochemical energy to regenerate NADPH from NADP^+^ via photosynthetic electron-transfer reactions. Because cyanobacteria produce several aromatic secondary metabolites, they must possess active enzymatic pathways to transform those substances according to the cellular requirements. Nevertheless, there is a lack of knowledge about the regulation of the biosynthesis of aromatic substances by cyanobacteria. In fact, very little is known about the mechanisms and consequences of the absence or overproduction of these substances in the cells of photoautotrophic bacteria.

Unfortunately, knowledge about the transport of these aromatic compounds through the cell walls and membranes of cyanobacteria is also lacking. Understanding these aspects seems to be crucial regarding the possibilities of the biotransformation and biodegradation of these chemicals by blue-green algae. The relevant data in the literature indicate that the biotransformation of large aromatic compounds, i.e. tricyclic and polycyclic molecules, occurs to a much lower extent than the biotransformation of mono- or even diaromatic systems. Access to the interior of cyanobacterial cells is probably hindered by increasing the number of aromatic residues, and this effect decreases the ability of cyanobacteria to transform aromatic compounds. As cyanobacteria have become one of the most attractive hosts for biotechnological production, due to their intensive metabolism, high proliferative ability and relative ease of genetic manipulation, a thorough understanding of the intracellular transformations of aromatic derivatives by these microorganisms is urgently needed.
